# The Emerging Role of Scalogram-Based Convolutional Neural Network in the Diagnosis of Epileptic Seizures

**DOI:** 10.3390/brainsci11111530

**Published:** 2021-11-18

**Authors:** Evanthia Bernitsas

**Affiliations:** Multiple Sclerosis Center, Wayne State University School of Medicine, Detroit, MI 48201, USA; ebernits@med.wayne.edu

Epilepsy, a common disorder affecting 1–2% of the population, can significantly impact a person’s quality of life and can lead to disability or even death. Current state-of the-art approaches include the use of EEG and MRI scans; however, in recent years, big advances have been made in the field of artificial intelligence. The use of deep learning networks, a form of machine learning, has been applied successfully over the last decade, as deep learning mimics the abilities of the human to identify, analyze, learn and solve complex problems. Deep Learning networks have been applied in a wide range of situations, ranging from cancer detection and COVID-19 diagnosis to neuroimaging and neuro-rehabilitation. Using deep learning networks is an efficient way to perform EEG signal processing for the detection of epilepsy [1]. It allows for an automated classification of features, and subsequently, it can decrease time commitment, effort and human error-associated processing. Convolutional Neural Network (CNN) is a deep learning algorithm that has been successfully used in a variety of machine learning applications. Its utilization has been substantially growing, and its contribution in the detection of epileptic seizures has become more significant [2].

In this issue of *Brain Sciences*, Drs. Turk and Ozerdem report on the development of a new approach that detects epileptic seizures. Using raw EEG data as an input and applying continuous wavelet transform (CWT), frequency-time scalograms images are obtained. These 2D images were resized to give the designed CNN input. The CNN, a multi-layered structure, takes an input image, learns the properties of the images, assesses the importance of various components of the image and differentiates one from another. The learning ability of the model is evaluated using a dataset of five EEG records found in the popular and most widely used University of Bonn database: the first two from healthy individuals (A and B) and the other three from patients with epilepsy (C, D and E, with only E recording ictal activity). A variety of parameters, such as sensitivity, specificity, accuracy and f-score are used to assess the model’s performance. Finally, the classification performance of this model is compared with the performance of a variety of other methods presented in relevant publications, using multi-comparisons, with all combinations of the EEG signals obtained and compared. The results confirmed that scalogram-based CNN processing of raw EEG signals provides superior performance in classification accuracy compared with different other methods used in studies published previously. An important finding of the study is that the accuracy of the proposed method is even higher with higher data set diversity. 

The proposed model showed an excellent learning ability, being able to learn the features of the 2D scalogram images and to differentiate between their specific features. Its superior classification accuracy compared with a large number of various studies using different algorithms is the major strength of this proposed model. In the highest data set diversity (A-B-C-D-E), the classification accuracy was 93.6% compared with 87.2% of other methods. In the A-D-E set, the accuracy was 99% compared with 95.67% and 98.47% of other algorithms. As epilepsy is a chronic, complex disease that requires long-term treatment and imposes limitations on leisure activities and employment, diagnostic accuracy is of paramount importance. Taking into consideration the high rate of about 23% of epilepsy misdiagnosis and the fact that 26% of children referred to epilepsy centers with “refractory epilepsy” found not to have epilepsy, high diagnostic accuracy is a feature of critical significance [3,4]. 

Additional strengths of this study include the input of raw, complete EEG data that contain a wealth of information rather than inputting only certain features resulting in losing meaningful data. Another advantage of the study is the use of CWT that allows for the analysis of high- and low-frequency information in the time series in contrast to short-time Fourier transform where all of the frequency information is analyzed at the same time-frequency resolution. In this study, no size-reduction methods were used and all classes were compared. CWT allows for 2D representation of the images, which offers additional advantages, such as containing the right amount of information, and required a reasonable but not excessive number of computational resources [5]. Even though the physician’s involvement and time invested were not recorded, this study is important for proposing a novel method that can improve the detection and diagnosis of epilepsy, in a cost-effective and reliable way.

This work by Turk and Ozerdem is very encouraging and represents a major step forward in the detection of epileptic seizures. As the deep learning models may be translated into everyday clinical practice in the near future, this proposed method may have significant applications and may eliminate challenges stemming from trial and error attempts, EEG artifacts and timing considerations. Moreover, it can be particularly valuable in the identification of early, atypical or not clear-cut cases of epilepsy and in the precise detection of non-epileptic seizures and rare epileptic disorders, targeting further interventions. Furthermore, it can assist with clinical decision making and guide future therapeutic approaches.
